# SUL-151 Decreases Airway Neutrophilia as a Prophylactic and Therapeutic Treatment in Mice after Cigarette Smoke Exposure

**DOI:** 10.3390/ijms22094991

**Published:** 2021-05-08

**Authors:** Lei Wang, Charlotte E. Pelgrim, Daniël H. Swart, Guido Krenning, Adrianus C. van der Graaf, Aletta D. Kraneveld, Thea Leusink-Muis, Ingrid van Ark, Johan Garssen, Gert Folkerts, Saskia Braber

**Affiliations:** 1 Division of Pharmacology, Utrecht Institute for Pharmaceutical Sciences, Faculty of Science, Utrecht University, 3584 CG Utrecht, The Netherlands; L.Wang@uu.nl (L.W.); C.e.Pelgrim@uu.nl (C.E.P.); A.D.Kraneveld@uu.nl (A.D.K.); A.Leusink@uu.nl (T.L.-M.); I.vanArk@uu.nl (I.v.A.); J.Garssen@uu.nl (J.G.); G.Folkerts@uu.nl (G.F.); 2 Sulfateq B.V., Admiraal de Ruyterlaan 5, 9726 GN Groningen, The Netherlands; d.swart@sulfateqbv.com (D.H.S.); g.krenning@sulfateqbv.com (G.K.); vandergraaf@citeq.nl (A.C.v.d.G.); 3 Cardiovascular Regenerative Medicine, Department of Pathology and Medical Biology, University of Groningen, University Medical Center Groningen, 9713 GZ Groningen, The Netherlands; 4 Institute for Risk Assessment Sciences (IRAS), Utrecht University, 3584 CM Utrecht, The Netherlands; 5 Nutricia Research, Department of Immunology, 3584 CT Utrecht, The Netherlands

**Keywords:** COPD, inflammation, oxidative stress, budesonide, neutrophils, SUL compound, mitochondria, PINK1, chromanol

## Abstract

Chronic obstructive pulmonary disease (COPD) caused by cigarette smoke (CS) is featured by oxidative stress and chronic inflammation. Due to the poor efficacy of standard glucocorticoid therapy, new treatments are required. Here, we investigated whether the novel compound SUL-151 with mitoprotective properties can be used as a prophylactic and therapeutic treatment in a murine CS-induced inflammation model. SUL-151 (4 mg/kg), budesonide (500 μg/kg), or vehicle were administered via oropharyngeal instillation in this prophylactic and therapeutic treatment setting. The number of immune cells was determined in the bronchoalveolar lavage fluid (BALF). Oxidative stress response, mitochondrial adenosine triphosphate (ATP) production, and mitophagy-related proteins were measured in lung homogenates. SUL-151 significantly decreased more than 70% and 50% of CS-induced neutrophils in BALF after prophylactic and therapeutic administration, while budesonide showed no significant reduction in neutrophils. Moreover, SUL-151 prevented the CS-induced decrease in ATP and mitochondrial mtDNA and an increase in putative protein kinase 1 expression in the lung homogenates. The concentration of SUL-151 was significantly correlated with malondialdehyde level and radical scavenging activity in the lungs. SUL-151 inhibited the increased pulmonary inflammation and mitochondrial dysfunction in this CS-induced inflammation model, which implied that SUL-151 might be a promising candidate for COPD treatment.

## 1. Introduction

Chronic obstructive pulmonary disease (COPD) is a lung disease primarily characterized by the presence of airflow limitation and inflammation, due to elevated inflammatory cells, especially neutrophils, in the lungs [1]. Cigarette smoke (CS) is one of the major causes of COPD, which is responsible for chronic inflammation and mitochondria dysfunction in the lungs [2,3]. It is well known that mitochondria are an important source of reactive oxygen species (ROS) [4]. Decreased adenosine triphosphate (ATP) and increased ROS production in dysfunctional mitochondria cause an imbalance in intracellular homeostasis [5]. Recent studies identify changes in lung cell mitochondria, including a reduction in mitochondrial biogenesis, changes in mitochondrial DNA (mtDNA), selective degradation of mitochondria, and substantial morphological defects, as contributors to COPD pathogenesis [6,7,8,9,10,11]. 

Glucocorticoids, β_2_-adrenoceptor agonists, and muscarinic receptor antagonists are the major current therapy for COPD, which can reduce symptoms and/or exacerbations, but do not specifically target oxidative stress, nor do they diminish chronic respiratory inflammation and COPD progression or mortality in all patients [12,13]. A large case–control study indicated that treatment of COPD with a long-acting β_2_-agonist or a long-acting muscarinic antagonist was associated with a 50% higher risk of serious cardiovascular complications, including patients that had no cardiovascular disease history [14]. In addition, a large group of COPD patients have a poor response to glucocorticoids or are completely resistant [15]. Oxidative stress causes a reduction in histone deacetylase-2 (HDAC-2) activity and has been implicated as an important cause of steroid resistance in COPD [16,17]. Therefore, there is an urgent need for the development of novel therapies that effectively suppresses chronic inflammation in COPD patients.

Recently, a novel class of pharmacological compounds, 6-hydroxychromanols, were developed, which are also called SUL compounds. SULs are water-soluble Trolox-derivatives and accumulate in the mitochondria, displaying antioxidant and mitoprotective properties by alleviating ROS production and preserving ATP production [18]. Interestingly, SUL-121 (6-hydroxy-2,5,7,8-tetramethylchroman-2-yl (piperazin-1-yl) methanone), a racemic mix of SUL compounds dose-dependently reduced LPS-induced airway neutrophilia and airway hyperresponsiveness in guinea pigs. Furthermore, it also inhibited the cigarette smoke-induced interleukin 8 (IL-8) release accompanied by a decreased cellular ROS production in human airway smooth muscle cells [19]. 

**Figure 1:**
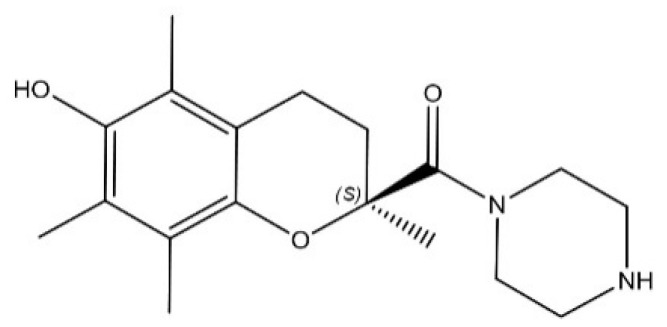
Chemical structure of SUL-151.

In this study, the efficacy of SUL-151 (Figure 1), the s-enantiomer of SUL-121, was examined in a prophylactic and therapeutic setting in mice triggered by CS to provoke oxidative stress and neutrophilic inflammation. Budesonide was used as a standard therapy to compare with the effectiveness of SUL-151 under the same conditions. Moreover, the mode of action of SUL-151 was explored. SUL-151 suppressed the CS-induced lung inflammation and mitochondrial dysfunction in this study, which appears as a promising candidate for the treatment of COPD to be assessed in future (pre) clinical trials.

## 2. Results

### 2.1. Prophylactic Treatment of SUL-151 Prevents Pulmonary Inflammation in a CS Exposure Model 

SUL-151 concentrations in the lungs after 5 days of oropharyngeal administration to the lungs were on average 768.3 ± 101.2 pg/mg (ranged between 125 and 2264 pg/mg protein). The levels of SUL-151 in the serum were below the lower limit of detection (i.e., <5 pg/mL, data not shown).

**Figure 2:**
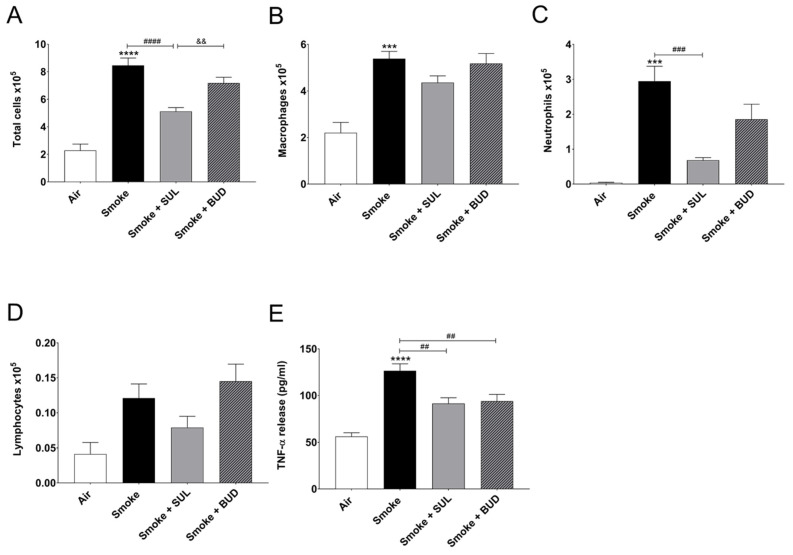
Prophylactic SUL-151 administration prevents pulmonary inflammation in a CS exposure model. Mice were exposed to CS for 5 days (twice/day) and received SUL-151 or budesonide via oropharyngeal administration (once/day) 30 min before the first-time smoke exposure during these 5 days. On day 6, lungs were lavaged, and BALF was collected for total (**A**) and differential BALF cell counts, including macrophages (**B**), neutrophils (**C**), and lymphocytes (**D**), and TNF-α levels (**E**) were measured in BALF. Values are expressed as mean ± SEM. *** *p* < 0.001, **** *p* < 0.0001, smoke group compared to air group; ^##^ *p* < 0.01, ^###^ *p* < 0.001, ^####^ *p* < 0.0001, smoke + SUL group or smoke + budesonide (BUD) group compared to smoke group; ^&&^ *p* < 0.01, smoke + BUD group compared to smoke + SUL group. *n* = 4–8 mice/group.

CS exposure for 5 consecutive days significantly increased the number of total BALF cells (Figure 2A), macrophages (Figure 2B), neutrophils (Figure 2C), and BALF TNF-α level (Figure 2E), when compared to the air control group. SUL-151 administration for 5 days to the air-exposed animals did not significantly affect the number of neutrophils, lymphocytes, or TNF-α levels in BALF; however, it doubled the number of total cells in BALF, represented by an increase in macrophages (Supplementary data Figure S1).

SUL-151 prophylaxis decreased the CS-induced increase in total cells in BALF (Figure 2A), wherein specifically neutrophilic infiltration was blunted (Figure 2C). Budesonide did not significantly affect the CS-induced increase of BALF total cells and neutrophils (Figure 2A,C). SUL-151 and budesonide did not affect the CS-induced increase in the numbers of macrophages and lymphocytes in BALF (Figure 2B,D); however, both compounds did reduce the TNF-α levels in BALF (Figure 2E).

### 2.2. Prophylactic Treatment of SUL-151 Prevents Oxidative Stress in the Lungs of CS-Exposed Mice

**Figure 3:**
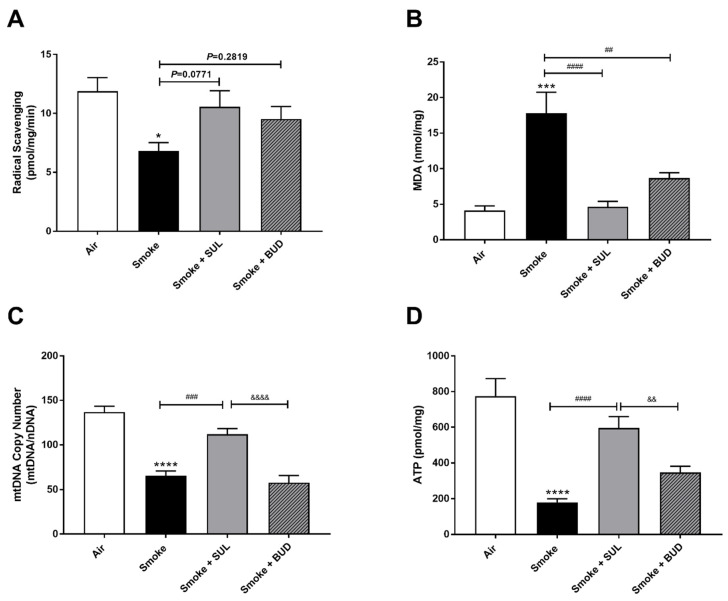
Prophylactic SUL-151 administration prevents oxidative stress in the lungs of a CS exposure model. Mice were exposed to CS for 5 days (twice/day) and received SUL-151 or budesonide via oropharyngeal administration 30 min before the first-time smoke exposure during these 5 days. On day 6, lung tissue was collected, and the radical scavenging activity (**A**), MDA concentration (**B**), mtDNA copy numbers (**C**), and ATP levels (**D**) were measured. Values are expressed as mean ± SEM. * *p* < 0.05, *** *p* < 0.001, **** *p* < 0.0001, smoke group compared to air group; ^##^ *p* < 0.01, ^###^ *p* < 0.001, ^####^ *p* < 0.0001, smoke + SUL group or smoke + budesonide (BUD) group compared to smoke group; ^&&^ *p* < 0.01, ^&&&&^ *p* < 0.0001, smoke + BUD group compared to the smoke + SUL group. *n* = 4–8 mice/group.

CS exposure for 5 consecutive days decreased the radical scavenging activity (Figure 3A) and increased the MDA levels (Figure 3B). In addition, a decrease of mtDNA (Figure 3C) and ATP (Figure 3D) was observed in the lung homogenates after CS exposure when compared to air-exposed mice. SUL-151 tended to increase the CS-induced decrease in radical scavenging capacity (*p* = 0.0771), but budesonide did not affect the CS-induced decrease in radical scavenging capacity (*p* = 0.2819) (Figure 3A), while both reduced the CS-induced increase in MDA in lung tissue (Figure 3B). SUL-151, but not budesonide, attenuated the loss of mitochondria by restoring the copy numbers of mtDNA (Figure 3C) and restored ATP production in lung tissue of CS-exposed mice (Figure 3D).

### 2.3. SUL-151 Reduces the Influx of Neutrophils in the BALF after the Development of CS-Induced Pulmonary Inflammation

**Figure 4:**
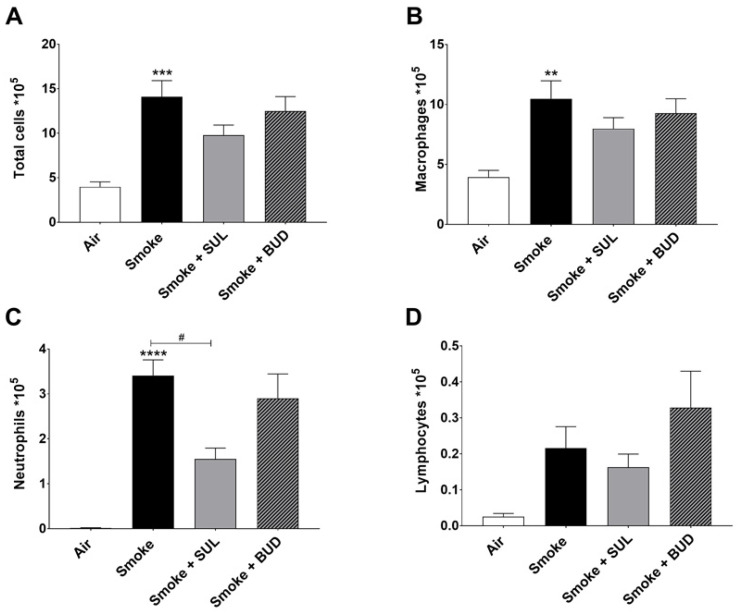
SUL-151 reduces the influx of neutrophils in the BALF after the development of CS-induced pulmonary inflammation. Mice were exposed to CS for 10 days (twice/day) and received SUL-151 or budesonide via oropharyngeal administration (once/day) 30 min before the first-time smoke exposure during the last 5 days of the CS exposure period. On day 11, lungs were lavaged, and BALF was collected for total (**A**) and differential BAL cell counts, including macrophages (**B**), neutrophils (**C**), and lymphocytes (**D**). Values are expressed as mean ± SEM. ** *p* < 0.01, *** *p* < 0.001, **** *p* < 0.0001, smoke group compared to air group; ^#^ *p* < 0.05, smoke + SUL group or smoke + budesonide (BUD) group compared to smoke group. *n* = 6–7 mice/group.

Based on the promising effects of SUL-151 prophylaxis in the CS exposure model, the effect of SUL-151 was further explored in a therapeutic setting. Mice were first exposed to CS for 5 days without therapy, followed by 5 days of CS exposure with the same therapies as described for the prophylactic approach. The number of total BALF cells (Figure 4A), macrophages (Figure 4B), and neutrophils (Figure 4C) were significantly increased after 10 days of CS exposure, compared to the air-exposed group, while a slight effect of CS exposure was observed on the number of lymphocytes in BALF. 

Both the treatment with SUL-151 and budesonide did not significantly affect the CS-induced increase in the number of total cells, macrophages, and lymphocytes in the BALF (Figure 4A,B,D). No differences were observed in TNF-α levels in BALF (data not shown). SUL-151, but not budesonide, was able to significantly reduce the CS-induced influx of neutrophils in the second set of 5 days CS exposure (day 10, Figure 4C) to a similar degree as in the prophylactic setting (Figure 2C). 

### 2.4. SUL-151 Reduces the Oxidative Stress in the Lungs after the Development of CS-Induced Pulmonary Inflammation 

**Figure 5:**
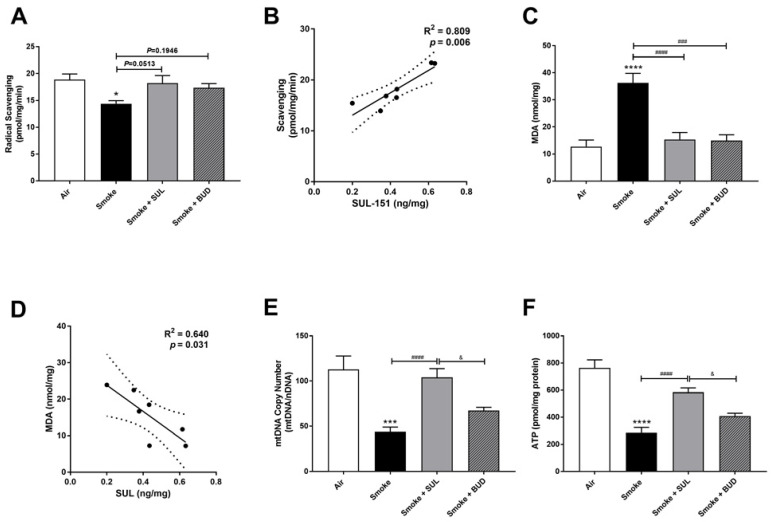
SUL-151 reduces the oxidative stress in the lungs after the development of CS-induced pulmonary inflammation. Mice were exposed to CS for 10 days (twice/day) and received SUL-151 or budesonide via oropharyngeal administration (once/day) 30 min before the first smoke exposure during the last 5 days of the CS exposure period. On day 11, lung tissue was collected, and the radical scavenging activity (**A**), MDA concentration (**C**), mtDNA copy numbers (**E**), and ATP levels (**F**) were measured. Correlation of radical scavenging activity and SUL-151 concentration in lung tissue (**B**) and the correlation of MDA and SUL-151 concentration in lung tissue (**D**) analyzed using Pearson r correlation test. Values are expressed as mean ± SEM. * *p* < 0.05, *** *p* < 0.001, **** *p* < 0.0001, smoke group compared to air group; ^###^ *p* < 0.001, ^####^ *p* < 0.0001, smoke + SUL group or smoke + budesonide (BUD) group compared to smoke group; ^&^ *p* < 0.05, smoke + BUD group compared to smoke + SUL group. *n* = 6–7 mice/group.

The radical scavenging activity was decreased (Figure 5A), and MDA levels were increased (Figure 5C) in the lungs after CS exposure. Moreover, the mtDNA copy numbers (Figure 5E) and the ATP levels (Figure 5F) were decreased in the lung homogenates 10 days after CS exposure. As observed in the prophylactic setting, SUL-151 tended to restore the CS-induced changes in the radical scavenging ability (*p* = 0.0513, Figure 5A), and both SUL-151 and budesonide decreased MDA levels in the lungs obtained from the mice of the therapeutic approach (Figure 5C). The increase in scavenging activity and the reduction of lipid peroxidation products observed in individual mice correlated to the concentration of SUL-151 in the lung homogenates (Figure 5B,D, respectively). Interestingly, SUL-151, significantly restored mtDNA copy numbers and ATP levels in the CS-exposed mice, while budesonide was not effective at restoring mtDNA or ATP levels (Figure 5E,F).

### 2.5. SUL-151 Inhibits the Increase in PINK1-Expression in the Lungs after the Development of CS-Induced Pulmonary Inflammation 

**Figure 6:**
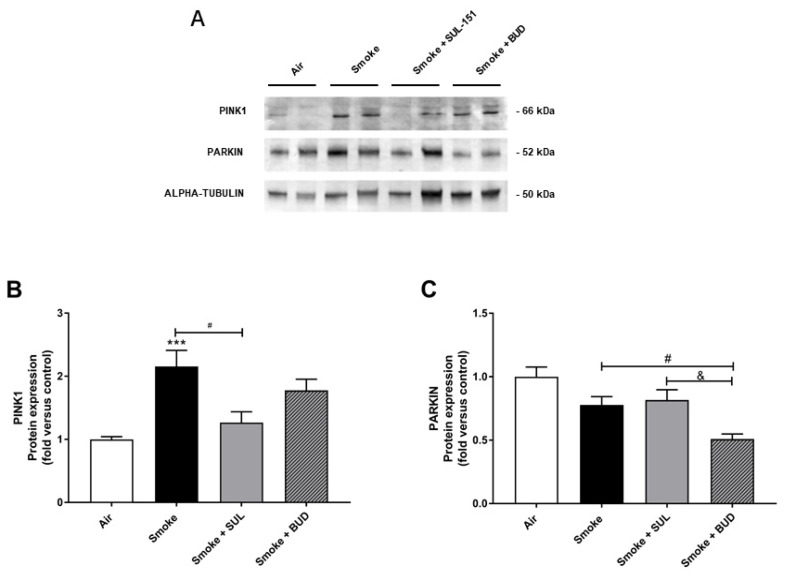
SUL-151 inhibited the increase in PINK1-expression in the lungs after the development of CS-induced pulmonary inflammation. Mice were exposed to CS for 10 days (twice/day) and received SUL-151 or budesonide via oropharyngeal administration (once/day) 30 min before the first smoke exposure during the last 5 days of the CS period. Western blot analysis for protein levels of PINK1, Parkin, and α-tubulin (**A**) was measured in the lung homogenates, and relative densities of the PINK1/α-tubulin (**B**) and Parkin /α-tubulin (**C**) were calculated. Values are expressed as mean ± SEM. *** *p* < 0.001, smoke group compared to air group; ^#^ *p* < 0.05, smoke + SUL group or smoke + budesonide (BUD) group compared to smoke group; ^&^ *p* < 0.05, smoke + BUD group compared to smoke +SUL group. *n* = 6–7 mice/group.

Parkin, a ubiquitin–protein ligase, and PTEN-induced putative kinase 1 (PINK1), a mitochondrial serine–threonine kinase, both exhibit protection against oxidative stress and act in a common mitophagy pathway [20,21]. The expression of PINK1 was significantly increased after 10 days of CS exposure. SUL-151, but not budesonide, significantly inhibited the CS-induced increase in PINK1 protein expression (Figure 6A,B). CS did not significantly change the Parkin protein expression in lung tissue (Figure 6A,C), while budesonide, but not SUL-151, further decreased the Parkin protein expression compared with the CS-exposed group.

### 2.6. SUL-151 Hardly Affects KC Levels in Lung Homogenates but Concentration-Dependently Inhibits IL8-Production in Human Bronchial Epithelial Cells 

**Figure 7:**
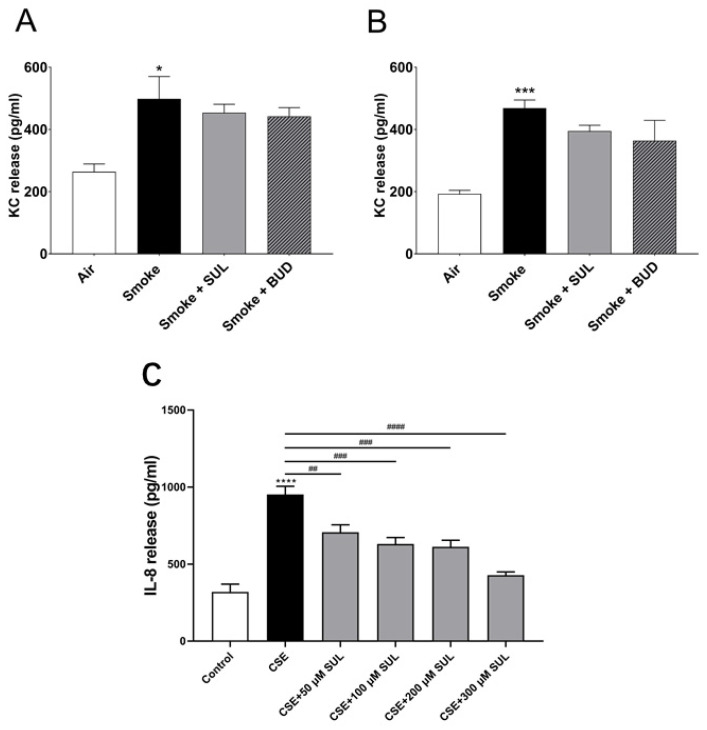
SUL-151 hardly affects KC levels in lung homogenates but concentration-dependently inhibits IL-8 production in human bronchial epithelial cells. Mice were exposed to CS for 5 days (twice/day) and received SUL-151 or budesonide via oropharyngeal administration 30 min before the first-time smoke exposure during these 5 days (**A**) or mice were exposed to CS for 10 days (twice/day) and received SUL-151 or budesonide via oropharyngeal administration (once/day) 30 min before the first smoke exposure during the last 5 days of the CS period (**B**). KC levels were measured in the lung homogenates via ELISA measurement. Human bronchial epithelial cells (16HBE cells), grown on 96-well plates, were preincubated with different concentrations of SUL-151 (50, 100, 200, and 300 µM) (24h) prior to CSE exposure for 24 h. Thereafter, IL-8 secretion was measured in the supernatants (**C**). Values are expressed as mean ± SEM. * *p* < 0.05, *** *p* < 0.001, **** *p* < 0.0001, smoke/CSE group compared to air/control group; ^##^ *p* < 0.01, ^###^ *p* < 0.001, ^####^ *p* < 0.0001, CSE group compared to the CSE + SUL group. *n* = 4–8 mice/group in vivo, *n* = 5, in vitro.

To find a link between the reduced oxidative stress and decrease in BALF neutrophil numbers, KC levels were measured in lung homogenates. Indeed, CS induced an increase in KC levels in the lungs. However, this CS-induced increase in KC levels was hardly affected by SUL-151 or budesonide as a prophylactic or therapeutic treatment (Figure 7A,B).

To further explore the effect of SUL-151 on IL-8 production, human bronchial epithelial (16HBE) cells were stimulated with cigarette smoke extract (CSE). IL-8 production was significantly enhanced after CSE exposure for 24 h. Surprisingly, SUL-151 concentration-dependently decreased the CSE-induced IL-8 production (Figure 7C). 

## 3. Discussion

COPD is one of the most common lung diseases worldwide, characterized by an accelerated loss of lung function [1]. Neutrophilic inflammation, a central feature of COPD, is closely associated with the severity of peripheral airway dysfunction in COPD [22]. Long-acting antimuscarinics or long-acting β_2_-adrenoceptor agonists, with or without inhaled glucocorticoids, are suggested by the COPD guidelines [23]. Clinical studies indicate that inhaled budesonide can be a good alternative for patients with acute exacerbation of chronic obstructive pulmonary disease [24]. These strategies reduce respiratory symptoms and exacerbations but do not affect the disease progression or suppress the sustained inflammation. In addition, most COPD patients respond poorly to high doses of inhaled or oral glucocorticoids. PDE4 inhibitors are new therapeutic options for COPD and a few new candidates, such as the dual PDE3/PDE4 inhibitor Ensifentrine, entered a phase 3 clinical trial, but none has yet reached the market. Therefore, there is an urgent need for the development of safe and effective new drugs for patients with COPD.

Recently, a novel class of pharmacological compounds was developed of which SUL-121 was one of the lead structures exhibiting promising mitoprotective effects due to the antioxidative capacities [25]. Our colleagues of the University of Groningen examined the pharmacological potential of SUL-121 in in vivo experimental airway inflammation models and showed that SUL-121 could prevent airway neutrophilia in BALF, airway hyperresponsiveness, and oxidative stress in the airways caused by LPS in a guinea pig model. SUL-121 also exhibited the capacity to inhibit the CS-induced IL-8 release in cultured human airway smooth muscle cells, which was associated with an inhibition of cellular ROS generation and a decrease in nuclear translocation of Nrf2 [19]. Based on these findings, we now explored the efficacy of the s-enantiomer of SUL-121, SUL-151, in a murine model of CS-induced lung inflammation. SUL-151 was administered into the airways for 5 days during CS exposure and the therapeutic possibilities were investigated by first exposing the mice to CS for 5 days without treatment followed by 5 days CS exposure with SUL-151 treatment. The efficacy was compared with the standard COPD treatment: the glucocorticoid budesonide. 

SUL-151 was administrated oropharyngeally to the lungs and was detected in the lung, which indicated that oropharyngeal administration is an effective route to target the lung in this model. The concentration of SUL-151 in the serum was lower than the detection limit, which might indicate that the compound remained and functioned in the lungs without leakage into the systemic compartment, and was completely metabolized in the lungs with no parent molecule present in the serum or was cleared at the endpoint of the study.

Neutrophilic inflammation is an important hallmark for COPD [22], which has been extensively associated with disease pathogenesis and progression [26]. As expected on the basis of earlier results with SUL-121 [19], SUL-151 significantly decreased the number of neutrophils in the BALF by more than 70% when used prophylactically and by ~50% when used therapeutically. In contrast, budesonide did not significantly decrease the number of neutrophils in the BALF, although the efficacy of this dose of budesonide (500 μg/kg) was confirmed in a murine asthma model [27] and in an LPS-induced acute lung injury model [28]. This dose seems rather ineffective in the CS-induced inflammation model and glucocorticoid insensitivity might be developed, as observed in the neutrophil influx. 

Due to the activation of inflammatory cells, during the inflammatory process in the lungs of COPD patients, oxidative stress is induced, which causes an oxidant/antioxidant imbalance [29]. Oxidative stress, including elevated ROS levels, is a major mechanism driving airway inflammation and airway damage in the pathophysiology of COPD [30,31]. A possible mechanism by which oxidants can cause lung injury is via lipid peroxidation [32]. MDA, a lipid peroxidation product, which is linked to the severity of COPD [33,34], was significantly increased after 5 and 10 days of CS exposure. Exhaled ethane, a marker for lipid peroxidation, is also elevated in COPD patients and correlates with FEV_1_ [35]. Moreover, serum MDA concentration correlates with FEV_1_ and disease severity in patients with COPD [36]. In our study, budesonide significantly decreased the CS-induced MDA increase in the lungs, which is in an agreement with the study of Zhang et al. [37], who showed that budesonide decreases MDA levels by scavenging free radicals and ROS in a bronchitis model with rats [37]. Compared to budesonide, SUL-151 inhibited the CS-induced MDA levels in the lungs and prevented the CS-induced decrease in ATP and mtDNA copy numbers, emphasizing that SUL-151 may influence the mitophagy pathway.

The observed decrease in ATP after CS exposure can be caused by damage to mitochondria, which is associated with lipid peroxidation, mitochondrial swelling, and disrupted mitochondrial respiratory chain [38,39]. The CS-induced decrease in ATP production and mtDNA copy numbers in the lungs was consistent with previous research findings [40,41]. mtDNA, as a specific genome of mitochondria, can present thousands of copies and form parts of the respiratory chain complex but are vulnerable due to the lack of protective histones and the capacity of DNA repairment [42]. CS is one of the main factors for oxidative stress in COPD, which can lead to mtDNA damage, mutation, and mitochondrial dysfunction in the lung [43]. We show here that, compared to budesonide, SUL-151 protects mitochondria in the lungs from CS-induced damages, as observed in the maintenance of both mtDNA copy numbers and ATP production.

The damaged mitochondria and mitochondrial dysfunction caused by CS might be related to mitophagy. Mitophagy is considered as a mechanism of selectively delivering the damaged mitochondria for lysosomal degradation, which is regulated by the PINK1-Parkin pathway. The PINK1–Parkin-dependent mitophagy might be involved in the pathogenesis of COPD [3,21]. In unhealthy mitochondria, the inner mitochondrial membrane becomes depolarized, which causes PINK1 to accumulate in the outer mitochondrial membrane. Increased mitophagy is related to the Parkin translocation to the damaged mitochondria, accompanied by PINK1 accumulation [11,44]. In our experiment, PINK1 accumulation was found after CS exposure, which was reduced by SUL-151 but not budesonide. Moreover, although Parkin levels were not significantly affected by CS exposure, budesonide, but not SUL-151, further decreased the level of Parkin. The decreased Parkin might indicate the less effective mitophagy, thus leading to more damaged mitochondria accumulated in the lung. 

To link the reduction in oxidative stress with the decreased number of neutrophils in the BALF, KC levels were measured in lung homogenates. Unfortunately, SUL-151 did not significantly reduce the CS-induced increase in KC levels. In contrast, the in vitro data on human bronchial epithelial cells showed that SUL-151 concentration-dependently inhibits IL-8 levels after CSE stimulation. Similar results were obtained with airway smooth muscle cells [19]. However, it has to be stressed that in vivo, the mice were exposed to CS twice a day and SUL-151 was only administered once, 30 min before the first CS exposure. It cannot be excluded that SUL-151 was more effective during the first CS challenge and inhibited KC production and hence the influx of neutrophils, while during the second CS exposure, SUL-151 was less effective since SUL-151 concentration dropped over time, leading to KC production and corresponding neutrophil influx.

This is supported by the fact that the influx of neutrophils was partly reduced by SUL-151 (50–70%). In our in vitro experiments, it was clearly demonstrated that SUL-151 almost completely decreased the CSE-induced IL-8 production in human bronchial epithelial cells. Based on these results, it is tempting to speculate that the effectiveness of SUL-151 might be further increased when administered twice daily.

As mentioned before, oxidative stress is proposed as the basis for the development of COPD following exposure to CS [45]. CS increases the burden of oxidants in the respiratory tract, either directly included in CS [46] or generated by inflammatory cells, depleting antioxidant defenses and injuring lung cells and beyond [11]. Although promising results of antioxidants have been highlighted by Fisher et al. (2015), this group of medicines in the treatment of COPD has not reached the market yet. SUL-151, however, as mentioned above, specifically prevents ROS production in the mitochondria, which may maintain the mitochondrial function and dampen subsequent inflammatory processes. Targeting oxidative stress in mitochondria might offer opportunities as a future therapy, especially since the consequent specific neutrophilic inflammation was tremendously suppressed. Such a pronounced reduction in an ongoing airway inflammation has not been demonstrated before with regular interventions, such as antioxidant treatments. Glucocorticoids are the most effective anti-inflammatory therapy, yet are relatively ineffective in COPD patients [47]. Various molecular mechanisms of glucocorticoid resistance have been characterized, which are associated with post-translational modifications of the glucocorticoid receptor. Due to the oxidative/nitrative stress, the activity and expression of HDAC-2 are noticeably inhibited, leading to a relative resistance to the anti-inflammatory actions of glucocorticoids [48]. Therefore, it would be interesting to further investigate whether an SUL-151 treatment or pretreatment with SUL-151 before glucocorticoid administration can treat COPD and prevent the development of glucocorticoid insensitivity, respectively. Due to the fact that the mode of action of SUL-151 is different from that of phosphodiesterase-4 inhibitors, combination therapies with this group of medicines may also be interesting [49].

**Figure 8:**
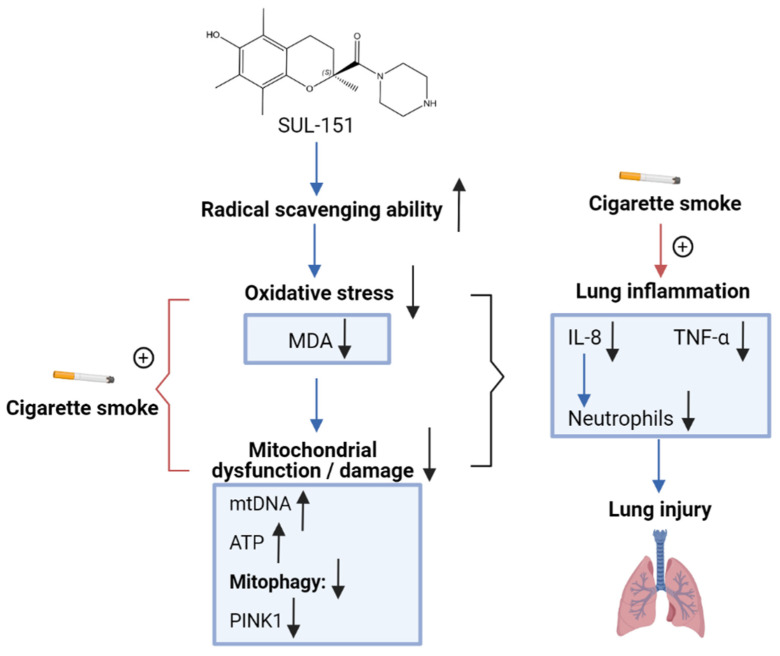
Schematic overview of the postulated mechanism of SUL-151 on CS-induced lung inflammation. SUL-151 has the ability to increase radical scavenging in the airways, possibly leading to a decrease in the CS-induced oxidative stress as measured by inhibition in MDA levels in lung homogenates. The decrease in oxidative stress might lead to a decrease in mitochondrial dysfunction/damage, as observed by the increase in mtDNA, the capacity to produce more ATP in the lungs, and the mitoprotective properties measured by a decrease in PINK1 expression. In this regard, the CS-induced oxidative stress and mitochondrial dysfunction can be inhibited by SUL-151, in turn leading to a decrease in CS-induced lung inflammation.

This study supports the hypothesis that SUL-151, a mitoprotective compound with modest antioxidant capacity, exert anti-inflammatory effects by normalizing oxidative stress (Figure 8 depicts a schematic overview of the postulated mechanism of SUL-151), and it might be a promising therapeutic option in the treatment of COPD. 

## 4. Materials and Methods

### 4.1. Animals

Specific-pathogen-free female Balb/c mice, 8–10 weeks old, were obtained from Charles River Laboratories and housed under standard conditions on a 12 h light/dark cycle in filter-topped Makrolon cages at the animal facility of the Utrecht University. Food and water were provided ad libitum and mice were randomly divided into experimental groups (*n* = 4–8 mice/group for the prophylactic approach and *n* = 6–7 mice/group for the therapeutic approach). All animal procedures described in this study were approved by the independent ethics committee for animal experimentation (the Ethical Committee of Animal Research of Utrecht University, Utrecht, the Netherlands) (DEC number: AVD243002016408) on 14th of March 2016 and were conducted in accordance with the governmental guidelines. 

### 4.2. Experimental In Vivo Procedures

**Figure 9:**
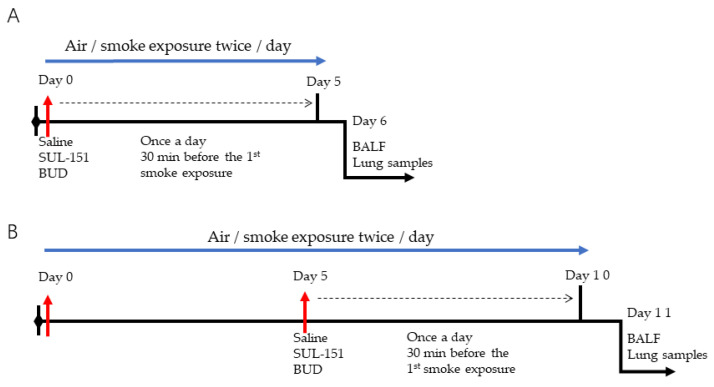
Schematic overview of the experimental design for the prophylactic treatment (**A**) and therapeutic treatment (**B**) of vehicle (saline), SUL-151, and budesonide (BUD) in the CS exposure model. The blue line represents the smoke exposure procedure (twice a day); the dotted line represents the saline, SUL-151, and BUD treatment (once a day, 30 min before the first round of smoke exposure).

In this study, two CS exposure experiments were conducted: (1) the prophylactic approach, in which SUL-151 (4 mg/kg), budesonide (500 μg/kg) [27], or vehicle (saline) was administered via the oropharyngeal route 30 min before the first CS exposure daily for 5 consecutive days to investigate the anti-inflammatory and antioxidative effects of SUL-151 (SUL-151 administration during the induction of the disease, Figure 9A) and (2) the therapeutic approach, in which mice were first exposed to CS for 5 days without treatment, followed by 5 days of CS exposure with the different therapies (once daily, 30 min before the first CS exposure) and a possible anti-inflammatory and antioxidative therapeutic approach of SUL-151 and associating potential mechanism were investigated (SUL-151 after induction of the disease, Figure 9B). 

Mice were exposed in whole-body chambers to air or to mainstream CS for 5 or 10 consecutive days (twice a day) using a peristaltic pump (SCIQ 232, Watson-Marlow 323, USA) at speed 35 rpm [50]. Research cigarettes (3R4F) were obtained from the Tobacco Research Institute (University of Kentucky, Lexington, Kentucky), and filters were removed before use. 

Mice were exposed to either CS or to ambient air twice daily with a minimum interval of 5 h using 4–6 cigarettes on day 1, 8–10 cigarettes on day 2, 12–14 cigarettes on day 3, and 14 cigarettes till the end of the smoke exposure period (two cigarettes/round). CS exposures were analyzed periodically for carbon monoxide (CO), ranging between 200 and 400 ppm. The mass concentration of cigarette smoke total particulate matter (TPM) was determined by gravimetric analysis of type A/E glass fiber filter (PALL life sciences, Mexico) [51]. The TPM concentration in the smoke exposure box generated by 14 cigarettes reached approximately 828 μg/L (828 ± 4.5 μg/L).

Mice were killed by an intraperitoneal overdose of pentobarbital approximately 18 h hours after the last air or smoke exposure. Blood was obtained by heart puncture and collected in Mini collect tubes (Greiner Bio-One, Alphen aan den Rijn, the Netherlands). Blood samples were centrifuged (14,000 rpm for 10 min) and serum was stored at −20 °C. Lungs were collected, snap-frozen in liquid nitrogen, and kept at −80 °C until further analyses. 

### 4.3. Bronchoalveolar Lavage Fluid (BALF) 

Directly after blood sample collection, the trachea was exposed, and a cannula was inserted in the trachea after making a small incision. BALF was collected by lung lavage with 1 mL pyrogen-free saline (0.9% NaCl, 37 °C), supplemented with protease inhibitors (Complete Mini, EDTA-free Protease Inhibitor Cocktail, Sigma-Aldrich, Zwijndrecht, the Netherlands). This step was repeated three times with 1 mL pyrogen-free saline. The BALF was centrifuged (400× *g*, 4 °C, 5 min) and the supernatant of the first mL was stored at −20 °C for ELISA measurement, and the pellets of the four lavages were pooled. Total numbers of BALF cells were counted using a Bürker-Türk chamber and differential BALF cell counts of macrophages, neutrophils, lymphocytes, and eosinophils were determined on cytospin preparations stained by Diff-Quik (Merz & Dade A.G., Düdingen, Switzerland). Differential cell counts were performed with at least 200 cells [52]. The first ml BALF was used for TNF-α measurements by ELISA (Mouse TNF-α ELISA kit, Thermo Fisher Scientific, Waltham, MA, USA) according to the manufacturer’s instructions.

### 4.4. Radical Scavenging Activity, Lipid Peroxidation Product Malondialdehyde (MDA), and ATP Measurements

Lung samples were homogenized in ddH_2_O using a TissueRuptor II (Qiagen, Hilden, Germany), followed by sonication at 20 kHz for 3 times 1 min (Sonopuls 2000, Bandelin, Berlin, Germany) and centrifugation at 14,000× *g* to pellet insoluble proteins. The supernatant was used to assess radical scavenging activity by ABTS radical decolorization [53], and lipid peroxidation by assessing the reactivity to thiobarbituric acid [19]. ATP content was determined using the ATP Determination Kit (Thermo Fisher Scientific, Waltham, MA, USA) according to manufacturer’s instructions. The total protein content of the supernatants was determined by DC Protein Assay (Bio-Rad, Hercules, CA, USA) and used for data normalization.

### 4.5. mtDNA Copy Number

Lung samples were homogenized in DNA isolation buffer (100 mM NaCl, 10 mM EDTA, 0.5% SDS in 20 mM Tris-HCl, pH 7.4) containing 50 U·mL^−1^ RNase I and 100 U·mL^−1^ proteinase K (both Fermentas, Waltham, MA, USA). After overnight incubation at 55 °C, total DNA was precipitated using 2-propanol. Aliquots of 10 ng DNA were amplified on a ViiA7 Real-time PCR system (Thermo Fisher Scientific, Waltham, MA, USA) using iTaq Universal SYBR Green Supermix (Bio-Rad, Hercules, CA, USA) and primers specific for mitochondrial DNA (MT-ND1; sense 5′-CGCCATAGCCTTCCTAACAT-3′, antisense 5′-ATGCCGTATGGACCAACAAT-3′) or nuclear DNA (NDUFA1; sense 5′-CCCCATGCTCTATCCATGTT-3′, antisense 5′-GCCATTTCTCTGCCTCTCAC-3′). Amplification was performed for 40 cycles of 15 s at 95 °C for denaturation, and 1 min at 60 °C for annealing and elongation. MtDNA copy number was calculated as mtDNA = 2 × 2 ^(Cq(NDUFA1)-Cq(MT-ND1)^.

### 4.6. Immunoblotting

Lung samples were homogenized in Radioimmunoprecipitation Assay (RIPA) buffer containing 0.5% proteinase inhibitor cocktail (Sigma-Aldrich, St. Louis, MO, USA) and 0.5% Halt™ phosphatase inhibitor (Thermo Fisher Scientific, Waltham, MA, USA) using a TissueRuptor II (Qiagen, Hilden, Germany), followed by sonication at 20 kHz for 3 × 30 s (Sonopuls 2000, Bandelin, Berlin, Germany) and centrifugation at 14,000× *g* to pellet insoluble proteins. The total protein content of the supernatants was determined by DC Protein Assay (Bio-Rad, Hercules, CA, USA). 10 µg of total protein per sample were loaded on mini-PROTEAN precast gradient gels (4–15% denaturing SDS–polyacrylamide gel; Bio-Rad, Hercules, CA, USA), separated by gel electrophoresis and blotted onto nitrocellulose membrane using the Trans-Blot Turbo System (Bio-Rad, Hercules, CA, USA) according to standard protocols. Blots were blocked with 5% bovine serum albumin in 20 mM Tris-HCl (pH 7.4) at room temperature for 30 min and incubated at 4 °C overnight with primary antibodies to PINK1 (BC100-494, Bio-Techne, Abingdon, UK), PARKIN (Santa Cruz Biotechnology, Dallas, TX, USA) or Tubulin (Sigma-Aldrich, St. Louis, MO, USA), all at a dilution of 1:500. Alkaline phosphatase-conjugated secondary antibodies and NBT/BCIP (Bio-Rad, VA, USA) were used for detection. Densitometric analysis was performed using Totallab 120 (Nonlinear Dynamics, Newcastle upon Tyne, England) [54]. 

### 4.7. Assessment of SUL-151 Levels in Serum and Lung 

Measurement of SUL-151 levels in the serum and in lung tissue homogenates was performed by chromatography and mass spectrometry. Serum or lung tissue homogenates were supplemented with acetonitrile and sonicated, followed by centrifugation at 14,000× *g* to liberate SUL-151 from the samples and pellet protein precipitates. Tissue samples were subjected to additional solid-phase extraction using an SPE Strata C-18 cartridge (100 mg, 55 µm, 70Å, Phenomenex, Torrance, CA, USA), prepared with 1 mL methanol, followed by 1 mL water. Analytes were eluted in acetonitrile:methanol (3:7 *v*/*v*). Analyte recovery was >70%. Liquid chromatography of the samples was performed on a 1260 Infinity HPLC device (Agilent Tech., Santa Clara, CA, USA) using a ZORBAX Eclipse AAA column (3.0 × 150 mm, particle size 3.5 µm) in a reversed-phase setup and a flow rate of 0.5 mL·min^−1^. Solvents consisted of methanol (6%): acetonitrile (4%) acetate and methanol (54%): acetonitrile (36%) in water with 0.1% ammonium for solvent A and B, respectively. MS/MS detection was performed on a QQQ 6460 mass spectrometer (Agilent Tech., Santa Clara, CA, USA). Detection was set for a quantifier ion (205.1, CE 25V) and a qualifier ion (190.1, CE 40V). Gas temperature for MS was set to 300 °C and flow was set to 6 L·min^−1^. Quantification of the samples was performed using an external standard for calibration. Limit of detection (LoD) and limit of quantitation (LoQ) were 5 pg·mL^−1^ and 17 pg·mL^−1^, respectively.

### 4.8. KC Measurement in the Lung Homogenates 

The lungs were homogenized in cold 1% Triton X-100 (Sigma-Aldrich)/PBS solution containing protease inhibitors (Complete Mini, EDTA-free Protease Inhibitor Cocktail, Sigma-Aldrich, Zwijndrecht, the Netherlands) using the Precellys 24 tissue homogenizer (Bertin Technologies, France). Thereafter, lung homogenates were centrifuged (15,000 rpm, 10 min, 4 °C) and the supernatant was collected. The protein concentration of the samples was measured by the Pierce BCA protein assay kit (Thermo Fisher Scientific, Waltham, MA, USA) according to the manufacturer’s instructions. Samples were diluted to 2 mg protein/mL and stored at −20 °C until further analyses. The KC levels were measured by ELISA (Mouse KC DuoSet ELISA kit, R&D system, Oxon, UK) according to the manufacturer’s instruction.

### 4.9. CSE Preparation and IL-8 Measurement in the Human Bronchial Epithelial Cells

CSE was freshly prepared just before each experiment by bubbling the CS of two 3R4F research cigarettes through 10 mL PBS without using the cigarette filters. Thereafter, the pH was adjusted to 7.4. The CSE preparation was standardized by measuring the optical density (OD) at 320 nm after filtering through a 0.22 μm filter using the spectrophotometer (UV mini-1240, SHIMADZU). The 100% CSE (OD~8) was diluted to a working concentration of 5% CSE and used in this experiment. 

An SV40-transformed and immortalized human bronchial airway epithelial cell line, 16HBE14o- (16HBE), was kindly provided by the University Medical Center Utrecht (Utrecht, The Netherlands). The cells were maintained and passaged in Minimum Essential Medium (MEM, Thermo Fisher Scientific) containing 10% inactivated fetal bovine serum (*v*/*v*) (Gibco, Brazil), 100 U/mL penicillin (Sigma-Aldrich, Zwijndrecht, the Netherlands), and 100 μg/mL streptomycin (Sigma-Aldrich, Zwijndrecht, the Netherlands). Cells (1 × 10^4^ cells /well) were seeded in 96-well plates till reaching 80–90% confluence and were pretreated with 50, 100, 200, 300 μM SUL-151 for 24 h before exposure to CSE for another 24 h at 37 °C in humidified atmosphere comprised of 95% air and 5% of CO_2_. The supernatant was collected, and IL-8 was measured by ELISA (Human IL-8 ELISA kit, R&D system, Oxon, UK) according to the manufacturer’s instruction.

### 4.10. Statistical Analysis

All results are presented as mean ± SEM. Differences between groups were statistically determined by one-way ANOVA, followed by Tukey’s multiple comparison post hoc test. The difference was considered statistically significant at *p* < 0.05. Pearson’s correlation test was conducted for analyses of correlation. All statistical analyses were conducted using GraphPad Prism (version 8.0).

### 4.11. Patents

Sulfateq B.V. holds preexisting intellectual property rights related to the work reported in this manuscript, especially PCT/EP2015/063579. No additional intellectual property claims resulted from the reported work.

## Data Availability

Raw data are stored at Utrecht University. The dataset used and/or analyzed during the current study are available from the corresponding author on reasonable request.

## Supplementary Materials

The following are available online at https://www.mdpi.com/article/10.3390/ijms22094991/s1, Figure S1: The effects of SUL-151 on pulmonary inflammation in the air-exposed group.
