# Supplementary figures and images for: SUL-151 Decreases Airway Neutrophilia as a Prophylactic and Therapeutic Treatment in Mice after Cigarette Smoke Exposure
Source: Int J Mol Sci. 2021 May 8;22(9):4991. doi: 10.3390/ijms22094991 (PMC8125869; doi:10.3390/ijms22094991)

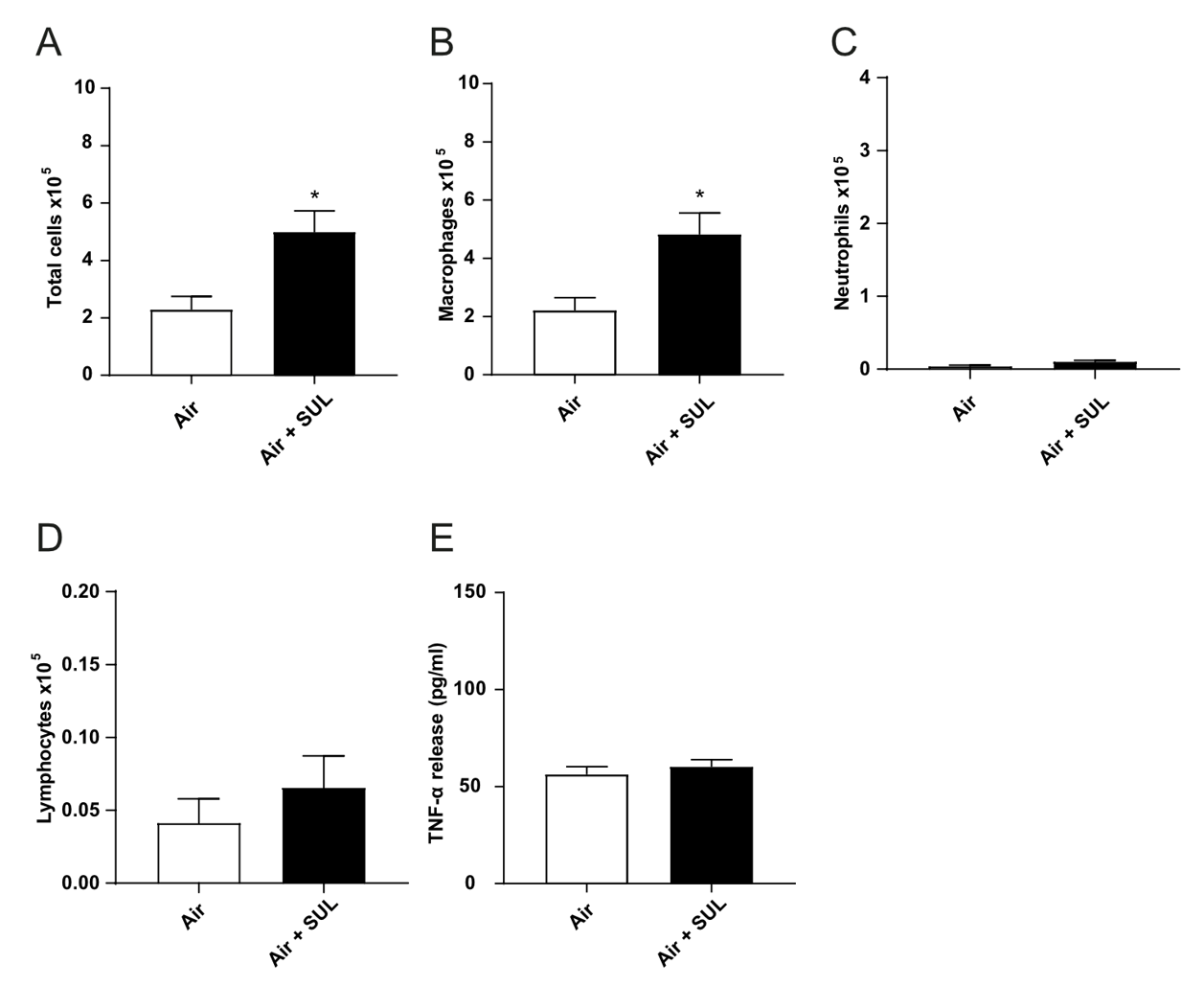

Supplement: Supplementary file 1 [file ijms-22-04991-s001.zip › Supplementary data 1.tif]
